# ABCA1 and cholesterol transfer protein Aster-A promote an asymmetric cholesterol distribution in the plasma membrane

**DOI:** 10.1016/j.jbc.2022.102702

**Published:** 2022-11-14

**Authors:** Fumihiko Ogasawara, Kazumitsu Ueda

**Affiliations:** Institute for Integrated Cell-Material Sciences (WPI-iCeMS), Kyoto University, Kyoto, Japan

**Keywords:** ABCA1, Aster-A (GramD1a), cholesterol flop, asymmetric transbilayer cholesterol distribution, PM–ER contact site, intracellular cholesterol homeostasis, BSA, bovine serum albumin, cDNA, complementary DNA, DMEM, Dulbecco’s modified Eagle’s medium, ER, endoplasmic reticulum, FBS, fetal bovine serum, HBSS, Hanks' balanced salt solution, HDL, high-density lipoprotein, HEK293, human embryonic kidney 293 cell line, IgG, immunoglobulin G, IPM, inner leaflet of the PM, LDL, low-density lipoprotein, MβCD, methyl-β-cyclodextrin, MEM, minimum essential medium, OPM, outer leaflet of the PM, PEcs, PM–ER contact site, PFO, perfringolysin O, PM, plasma membrane, SCAP, SREBP cleavage–activating protein, SMase, sphingomyelinase, SREBP, sterol regulatory element–binding protein, TIRF, total internal reflection fluorescence

## Abstract

Cholesterol is a major and essential component of the mammalian cell plasma membrane (PM), and the loss of cholesterol homeostasis leads to various pathologies. Cellular cholesterol uptake and synthesis are regulated by a cholesterol sensor in the endoplasmic reticulum (ER). However, it remains unclear how changes in the cholesterol level of the PM are recognized. Here, we show that the sensing of cholesterol in the PM depends on ABCA1 and the cholesterol transfer protein Aster-A, which cooperatively maintain the asymmetric transbilayer cholesterol distribution in the PM. We demonstrate that ABCA1 translocates (flops) cholesterol from the inner leaflet of the PM to the outer leaflet of the PM to maintain a low inner leaflet cholesterol level. We also found that when inner cholesterol levels were increased, Aster-A was recruited to the PM–ER contact site to transfer cholesterol to the ER. These results suggest that ABCA1 could promote an asymmetric cholesterol distribution to suppress Aster-A recruitment to the PM–ER contact site to maintain intracellular cholesterol homeostasis.

The cholesterol transporters ABCA1 and ABCG1 are essential for reverse cholesterol transport, a pathway through which cholesterol in peripheral tissues is delivered to the liver. ABCA1 transfers cholesterol and phosphatidylcholine to apoA-I, a lipid acceptor in blood, to generate high-density lipoprotein (HDL) (1), and ABCG1 exports cholesterol to HDL (2). ABCA1 is ubiquitously expressed in the body, and HDL generation is the only pathway for reverse cholesterol transport; thus, ABCA1 deficiency causes severe hypercholesterolemia or Tangier disease (3, 4, 5). Moreover, mutations in ABCA1 were found in patients with chronic myelomonocytic leukemia, suggesting that ABCA1 exerts tumor suppressor functions (6). Regarding *in vivo* studies, the knockout of ABCA1/G1 enhances macrophage inflammatory responses (7, 8, 9), and the tissue-specific knockout of ABCA1/G1 or ABCA1 has characteristic effects, including autoimmune activation in dendritic cells (10), impaired diet-induced obesity in adipose tissue (11), and less phagocytosis in astrocytes (12). It has been considered that these phenotypes are caused by excessive cholesterol accumulation because of defective cholesterol export. We recently reported that ABCA1 not only exports cholesterol but also translocates (flops) it from the inner leaflet of the PM (IPM) to the outer leaflet of the PM (OPM) (13, 14, 15). IPM cholesterol is maintained at around 3 mol% in various cell lines, whereas OPM cholesterol is 30∼50 mol% (16). This asymmetric cholesterol distribution allows cholesterol to function as an intramembrane signaling molecule (13, 17), but its physiological importance is not fully understood.

Cholesterol is synthesized in the endoplasmic reticulum (ER) and is also taken up as low-density lipoprotein (LDL) *via* LDL receptor. Cholesterol synthesis and uptake are regulated by sterol regulatory element–binding protein (SREBP) and SREBP cleavage–activating protein (Scap) (18). The SREBP–SCAP system is controlled by the ER cholesterol level; 5 mol% is the threshold that activates or deactivates the SREBP–SCAP system depending on the cellular cholesterol level.

In addition, cholesterol is a major component of the PM, which stores 60∼90% of total cholesterol in the cell (19, 20). It has been reported that Aster proteins (GRAMD1s) localized at the ER are recruited to the PM–ER contact site (PEcs) upon an increase in the PM cholesterol level to transfer cholesterol from the PM to the ER (21, 22). Aster-B is mainly expressed in steroidogenic tissues in wildtype mice, and Aster-B knockout mice show low steroidogenesis because of low adrenal cholesterol ester storage (21). Aster-C is mainly expressed in the liver and testis, but its physiological function is unknown (23). Wang *et al.* (24) showed that liver-specific Aster-B/C silencing ameliorates fibrotic nonalcoholic steatohepatitis by decreasing the cholesterol accumulation in hepatocytes that causes the disease. These studies suggest that Aster-B/C contributes to cholesterol internalization in cholesterol-abundant tissues and to cellular cholesterol accumulation. While it has been reported that, like Aster-B/C, Aster-A is recruited to the PEcs upon an increase in the PM cholesterol level and is ubiquitously expressed, its physiological role remains unclear. Given the aforementioned reports, we hypothesized that the low IPM cholesterol level formed by ABCA1 allows Aster-A to respond to a local and temporal increase in the PM cholesterol level for cholesterol internalization, which enables the SREBP–SCAP system to immediately and constantly sense the PM cholesterol level.

## Results

**Figure 1:**
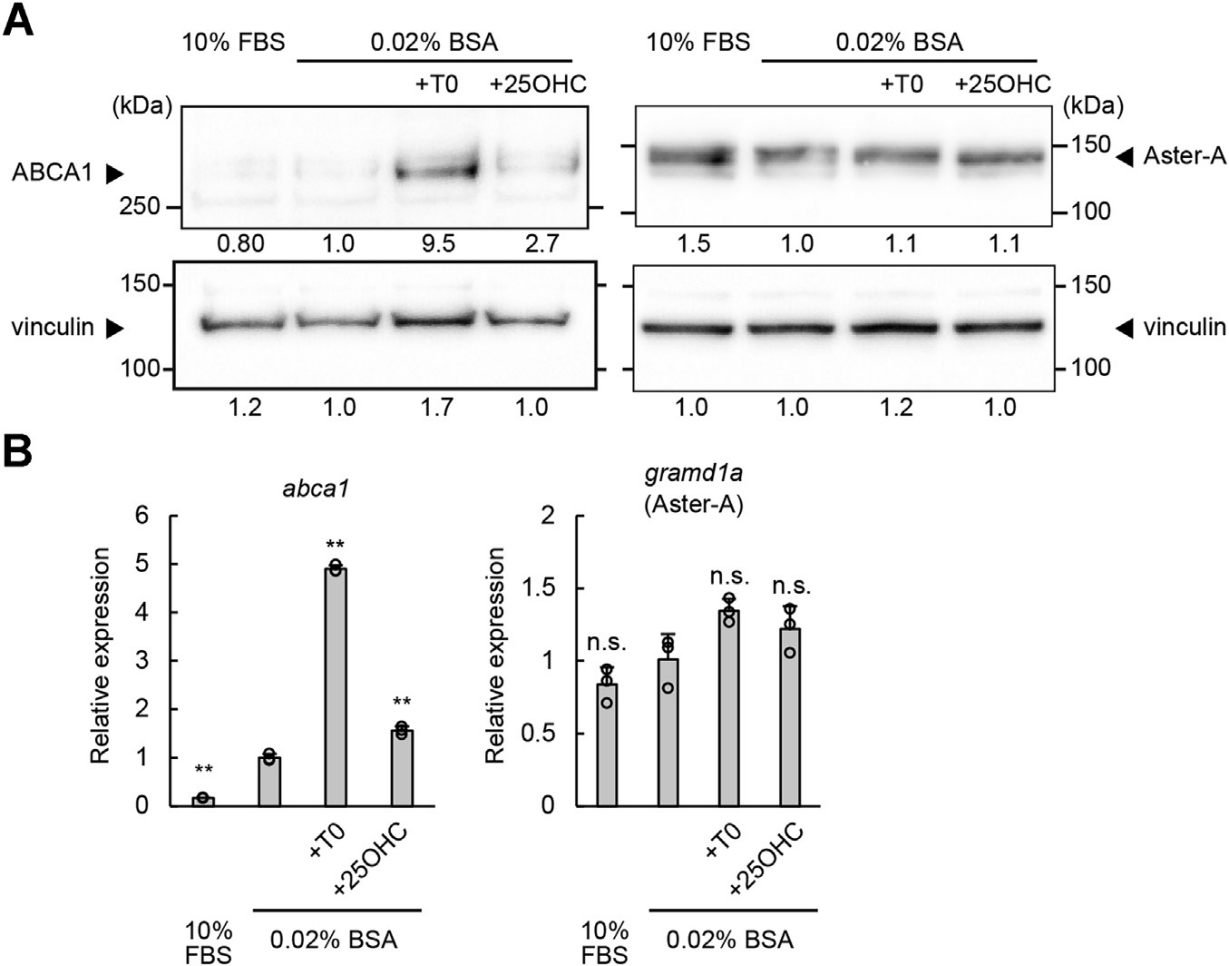
**Aster-A was not a transcriptional target of LXRs.** WI-38 cells, a normal human lung fibroblast *line*, were cultured in medium containing 10% FBS or 0.02% BSA with or without 10 μM T0901317 or 10 μM 25-hydroxycholesterol for 24 h. *A*, the protein expressions of Aster-A, ABCA1, and vinculin, a loading control, were analyzed by Western blotting. The anti-Aster-A antibody correctly recognized Aster-A (Fig. S1). The relative band intensities to the control (0.02% BSA) are shown below each band. *B*, the mRNA expressions of *gramd1a* (Aster-A) and *abca1* were analyzed by qPCR. Mean values are shown with SD. ∗∗*p* < 0.001 *versus* 0.02% BSA. n = 3. BSA, bovine serum albumin; FBS, fetal bovine serum; LXR, liver X receptor; ns, not significant; qPCR, quantitative PCR.

*Gramd1b* gene, which codes Aster-B, is a direct transcriptional target of sterol-responsive liver X receptors, and Aster-B expression is induced in response to an increased cholesterol level in the cell (21). We discovered, however, that Aster-A expression was induced by neither serum depletion nor liver X receptor agonists (Fig. 1), indicating that the expression level of Aster-A is not changed by the cellular cholesterol level. Considering its ubiquitous expression (21), Aster-A is expected to have a different role in systemic cells from Aster-B, which incorporates cholesterol mainly in cholesterol-abundant tissues.

**Figure 2:**
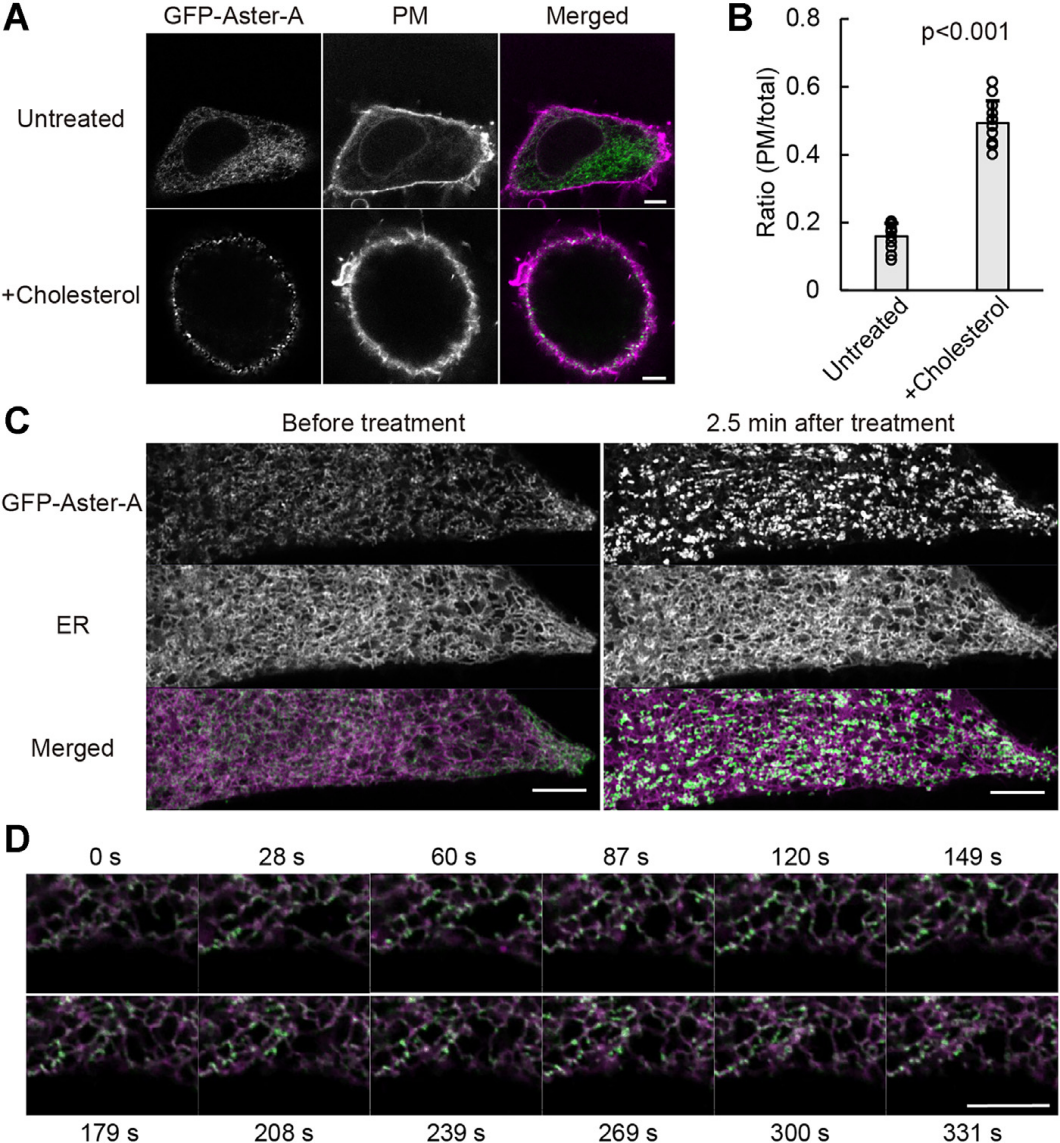
**Aster-A was recruited to the PEcs by loading cholesterol.***A*, HeLa/GFP-Aster-A cells were treated with or without 0.4 mM MβCD–cholesterol complex for 5 min, fixed with 4% paraformaldehyde, and observed by confocal microscopy. The PM was stained with CellMask *Deep Red*. The scale bars represent 5 μm. *B*, the ratio of GFP Aster-A on the PM to that in the total cell area is shown. Mean values are shown with SD. *p* < 0.001 *versus* untreated. n = 12 cells. *C*, images of HeLa/GFP-Aster-A cells transfected with mCherry-KDEL (an ER marker) and taken using AiryScan (*left*). Images taken 2.5 min after a final concentration of 0.4 mM MβCD–cholesterol complex was added (*right*). The scale bars represent 5 μm. *D*, representative images of GFP-Aster-A movement after cholesterol loading at a low concentration (0.1 mM) at each time point in Video S2. The scale bar represents 5 μm. ER, endoplasmic reticulum; MβCD, methyl-β-cyclodextrin; PEcs, PM–ER contact site; PM, plasma membrane.

To investigate the Aster-A function, we established HeLa cells stably expressing GFP-Aster-A. Western blotting showed a band that was shifted to a higher molecular weight than expected (108 kDa), but the band of endogenous Aster-A was also shifted higher (Fig. S2). The band of a C-terminal deletion mutant was also shifted, but that of the Gram domain was not. Thus, some structures or modifications in amino acids 256 to 546 including the Aster domain might affect the mobility in electrophoresis. We therefore concluded that GFP-Aster-A was correctly expressed. Next, the localization of Aster-A was examined by confocal microscopy. As reported (21), Aster-A was diffused throughout the ER and showed a peripheral dot-like distribution overlapping CellMask Deep Red (Thermo Fisher Scientific), a PM marker, by cholesterol loading using the methyl-β-cyclodextrin (MβCD)–cholesterol complex (Fig. 2*A*). The mean values of the ratio of GFP Aster-A on the PM to that in the total cell area with and without the cholesterol loading were 0.49 ± 0.07 and 0.16 ± 0.04, respectively (Fig. 2*B*). Furthermore, the movement of GFP-Aster-A on the ER network near the bottom of the cell was observed by high-resolution microscopy (Fig. 2*C* and Video S1). After the cholesterol loading, GFP-Aster-A molecules immediately showed a dot-like distribution in the ER network, where they hardly moved for a few minutes. The dot-like distribution was localized near the PM in Figure 2*A*, indicating that Aster-A was recruited to the PEcs by cholesterol loading. However, when the concentration of the cholesterol loading was low, the dot-like distribution of GFP-Aster-A was dynamic, repeatedly appearing and disappearing (Fig. 2*D* and Video S2). The cholesterol level of the PM does not significantly increase under physiological conditions except for some tissues or cells, such as the liver or macrophages. Thus, these results suggest that Aster-A diffuses on the ER and monitors the cholesterol level of the PM by changing the length of time it localizes at the PEcs.

**Figure 3:**
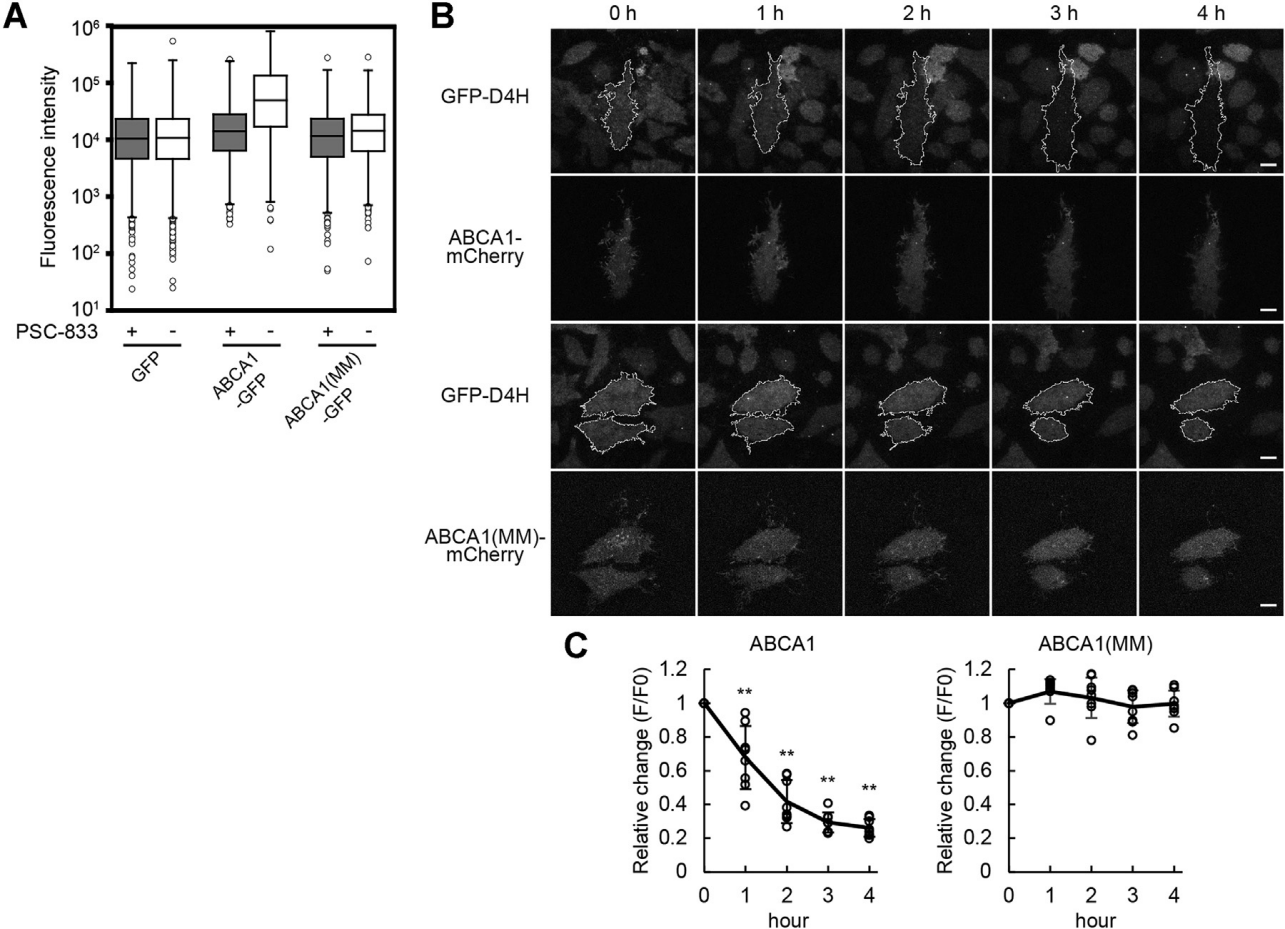
**ABCA1 flops cholesterol in the PM in HeLa cells.***A*, HeLa cells transfected with GFP, ABCA1-GFP, or ABCA1(MM)-GFP were cultured in medium containing 10% FBS and 2.5 μM PSC-833. On the following day, the cells were incubated in serum-free medium with or without PSC-833 for 2 h, and Alexa647-PFO-D4 binding to the cells was analyzed by flow cytometry. Fluorescence intensities of Alexa647-PFO-D4 in GFP-positive cells are shown. The flow cytometry plots are shown in Fig. S4. *B*, HeLa/GFP-D4H cells were transfected with ABCA1-mCherry or ABCA1(MM)-mCherry in medium containing 10% FBS and 5 μM PSC-833. After the medium was changed to serum-free medium, images were acquired every hour by TIRF microscopy. The *white outlines* indicate the regions of the cell expressing ABCA1-mCherry or ABCA1(MM)-mCherry. The scale bars represent 10 μm. *C*, relative changes in the GFP fluorescence intensity. *Solid lines* indicate mean values. Error bars indicate SD. ∗∗*p* < 0.001 *versus* ABCA1(MM)-expressing cells. n = 8 to 9 cells. FBS, fetal bovine serum; PM, plasma membrane; TIRF, total internal reflection fluorescence.

Previous reports show that Aster proteins sense the cholesterol concentration of the PM using their Gram domains, which bind to membranes containing cholesterol above a certain concentration (21, 22). The Aster protein structure shows long disordered regions between the Gram domain and Aster domain, which allows the Gram domain to freely bind to IPM cholesterol. We reported that ABCA1 flops cholesterol (13, 14), suggesting ABCA1 suppresses Aster-A recruitment to the PEcs. To show that ABCA1 flops cholesterol, we performed flow cytometry (Fig. 3*A*) using the Alexa Fluor 647–labeled D4 domain of perfringolysin O (PFO), which is the cholesterol-binding domain of pore-forming cytolysin (Fig. S3). GFP, ABCA1-GFP, or an ATP hydrolysis–deficient mutant, ABCA1(MM)-GFP, was expressed in HeLa cells. Because ABCA1 exports cholesterol to apoA-I, which is a lipid acceptor in blood and abundant in fetal bovine serum (FBS), an ABCA1 inhibitor (PSC-833) (25) was added at the time of the transfection. Before the observation, the cells were incubated in serum-free medium for 2 h to make ABCA1 flop cholesterol but not transfer the cholesterol onto apoA-I. In the cells expressing ABCA1-GFP, Alexa647-PFO-D4 binding increased with an increase in the expression level of ABCA1-GFP (Fig. S4), and the median of the fluorescence intensity of Alexa647-PFO-D4 binding to ABCA1-GFP–positive cells without PSC-833 increased 3.5 times compared with the condition with PSC-833 (Fig. 3*A*). In contrast, in the cells expressing GFP or ABCA1(MM)-GFP, Alexa647-PFO-D4 binding had a negligible effect. These results showed that ABCA1 increased the OPM cholesterol level in HeLa cells. Furthermore, the IPM cholesterol level was examined by total internal reflection fluorescence (TIRF) microscopy using PFO-D4H, which has a higher affinity for cholesterol than wildtype PFO-D4 (26). To observe a change in the IPM cholesterol level, we established HeLa cells expressing a proper level of GFP-D4H in the cytosol, because the expression level of the cholesterol probe greatly affects its translocation to the PM (16). When the cells were transiently transfected with ABCA1-mCherry and cultured in the presence of PSC-833, GFP-D4H gradually dissociated from the PM of the cells expressing ABCA1-mCherry after removing PSC-833 to exert ABCA1 activity, but in other cells without ABCA1 expression, GFP-D4H stayed at the PM (Fig. 3*B*). The relative fluorescence intensity of GFP-D4H in cells expressing ABCA1 decreased to 0.26 ± 0.05 at 4 h, but that in cells expressing ABCA1(MM)-mCherry remained constant (Fig. 3*C*). The same assay performed with another HeLa/GFP-D4H clone showed a similar result, suggesting that the observations are not clone specific (Fig. S5).

**Figure 4:**
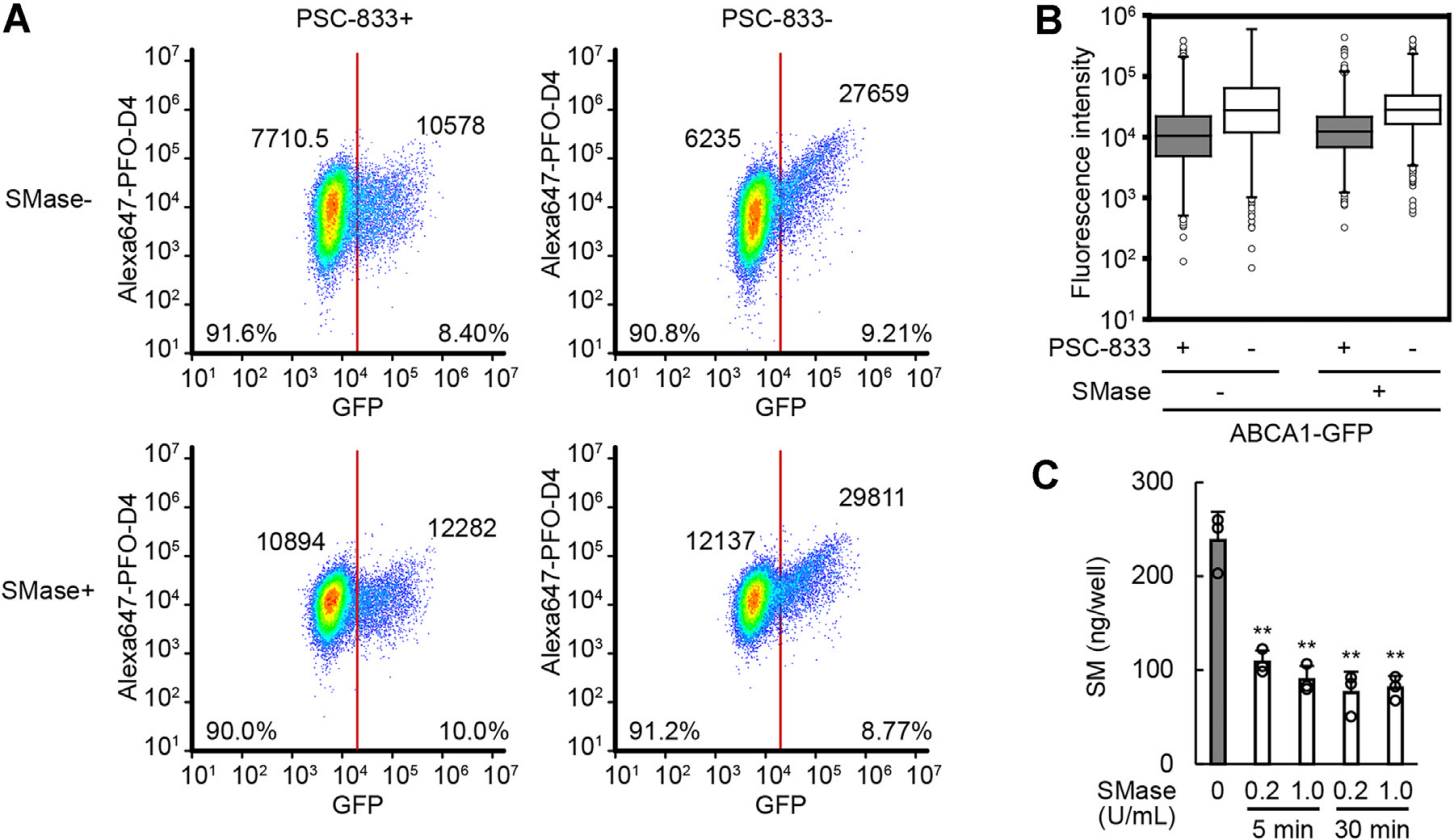
**ABCA1 increased the OPM cholesterol level even in sphingomyelin-depleted cells.***A* and *B*, HeLa cells transfected with ABCA1-GFP were incubated with or without PSC-833 or SMase in serum-free medium for 2 h. Alexa647-PFO-D4 binding to the cells was analyzed by flow cytometry. *A*, the plots were divided into GFP-negative and GFP-positive cells at a fluorescence intensity of 20,000, and the percentage of the plots and median values of the fluorescence intensities of Alexa647-PFO-D4 are shown. *B*, the fluorescence intensities of Alexa647-PFO-D4 in GFP-positive cells are shown. *C*, the amount of sphingomyelin in HeLa/GFP-Aster-A cells was measured after SMase treatment at the indicated conditions. Mean values are shown with SD. ∗∗*p* < 0.001 *versus* SMase 0 U/ml. n = 3. OPM, outer leaflet of the PM; PFO, perfringolysin O; SMase, sphingomyelinase.

When cholesterol interacts with sphingomyelin in the PM, PFO cannot bind to cholesterol (27). We examined whether ABCA1 increases the OPM cholesterol level even in sphingomyelin-depleted cells. Sphingomyelinase (SMase) was added at the same time PSC-833 was removed to exert ABCA1 activity. Although SMase treatment slightly increased Alexa647-PFO-D4 binding to cells lacking ABCA1 expression because cholesterol sequestered by sphingomyelin was released, ABCA1 increased the binding regardless of SMase treatment (Fig. 4, *A* and *B*). SMase rapidly depleted cell-surface sphingomyelin (Fig. 4*C*). The increase in Alexa647-PFO-D4 binding to the cells by ABCA1 confirmed the increase in the OPM cholesterol level. Therefore, these results suggest that ABCA1 flops cholesterol and decreases the IPM cholesterol level.

**Figure 5:**
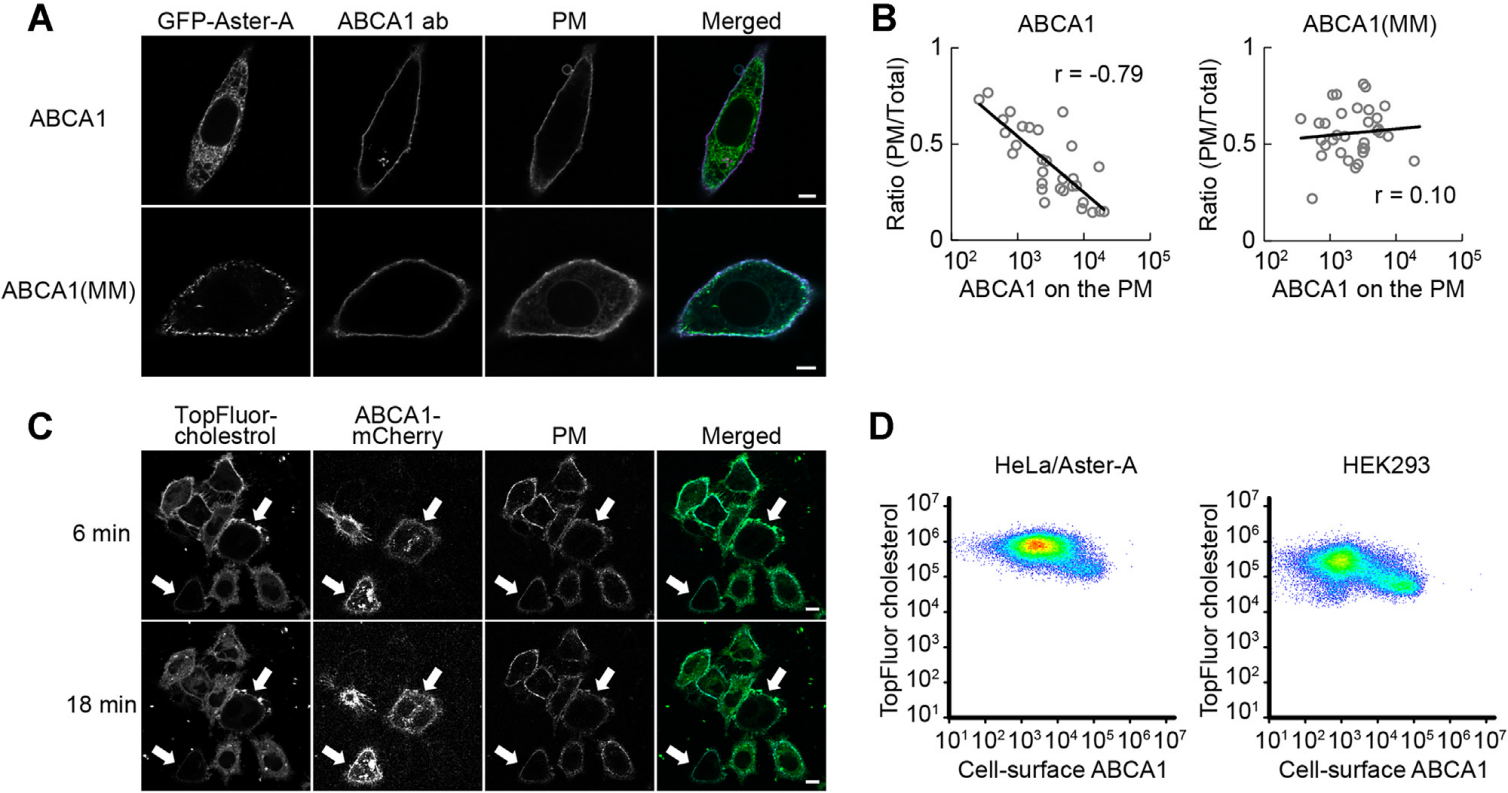
**The effect of ABCA1 on cholesterol-dependent Aster-A recruitment to the PEcs.***A*, HeLa/GFP-Aster-A cells transfected with ABCA1 or ABCA1(MM) were treated with 0.3 mM MβCD–cholesterol complex for 5 min and fixed with 4% paraformaldehyde. ABCA1 was stained with an antibody (ab) against the anti–extracellular domain of ABCA1 and observed by confocal microscopy. The PM was stained with CellMask *Deep Red*. The scale bars represent 5 μm. *B*, the ratio of GFP-Aster-A on the PM to that in the total cell area is plotted with the expression level of ABCA1 or ABCA1(MM) on the PM. Log-linear regressions (*solid lines*) and correlation coefficients (*r*) are shown. n = 30 to 33 cells. *C*, HeLa/Aster-A cells transfected with ABCA1-mCherry were treated with 0.2 mM MβCD–cholesterol complex mixed with TopFluor-cholesterol for 5 min and observed by confocal microscopy. *White arrows* indicate ABCA1-mCherry–expressing cells. The scale bars represent 10 μm. *D*, HeLa/Aster-A cells or HEK293 cells transfected with ABCA1 were treated with 0.2 mM MβCD–cholesterol complex mixed with TopFluor-cholesterol for 5 min and analyzed by flow cytometry. Cell-surface ABCA1 was measured with ABCA1 antibody. HEK293, human embryonic kidney 293 cell line; MβCD, methyl-β-cyclodextrin; PEcs, plasma membrane–endoplasmic reticulum contact site; PM, plasma membrane.

Next, we examined whether ABCA1 suppresses cholesterol-dependent Aster-A recruitment. ABCA1 or ABCA1(MM) was expressed in HeLa/GFP-Aster-A cells, which were fixed at 5 min after cholesterol loading, and the localization of GFP-Aster-A was observed by confocal microscopy (Fig. 5*A*). GFP-Aster-A in cells highly expressing ABCA1 was evenly diffused in the ER, but in cells expressing ABCA1(MM), it was recruited to the PEcs. The ratio of the fluorescence intensity in the PM to that in the total cell area was plotted with the ABCA1 expression level on the PM, which was measured using an antibody against the extracellular domain of ABCA1 (15) without permeabilization (Fig. 5*B*). The correlation coefficient between the ratio and the ABCA1 and ABCA1(MM) expression levels was −0.79 and 0.10, respectively. While ABCA1 is ubiquitously expressed in the body, the expression levels differ greatly among tissues and are highly regulated by intracellular cholesterol levels. Our data show that Aster-A recruitment to the PEcs is dependent on the expression level of ABCA1 over a concentration range of more than 100-fold, suggesting that ABCA1 regulates Aster-A recruitment to the PEcs in various tissues under different conditions. To visually confirm that ABCA1 suppresses Aster-A-mediated cholesterol internalization, we added TopFluor-cholesterol, which is cholesterol conjugated with a fluorescent dye, to the PM and observed the internalization in living cells. HeLa/Aster-A cells transiently expressing ABCA1-mCherry were treated with TopFluor-cholesterol mixed with the MβCD–cholesterol complex, incubated for 5 min, and observed by confocal microscopy (Fig. 5*C*). However, unexpectedly, the TopFluor-cholesterol fluorescence on the PM of ABCA1-mCherry–expressing cells was lower than in cells without ABCA1 expression, and the internalization of TopFluor-cholesterol was apparently slower. Indeed, the fluorescence intensity of TopFluor-cholesterol in cells expressing ABCA1 tended to be lower in a flow cytometry assay (Fig. 5*D*). This result was replicated in human embryonic kidney 293 (HEK293) cells, verifying that the phenomenon is not cell-type specific. The high OPM cholesterol level generated by ABCA1 might prevent cholesterol transfer from the MβCD–cholesterol complex to the PM. Following these results, we decided to apply another method to examine the effect of ABCA1 on cholesterol-dependent Aster-A recruitment to test our hypothesis.

**Figure 6:**
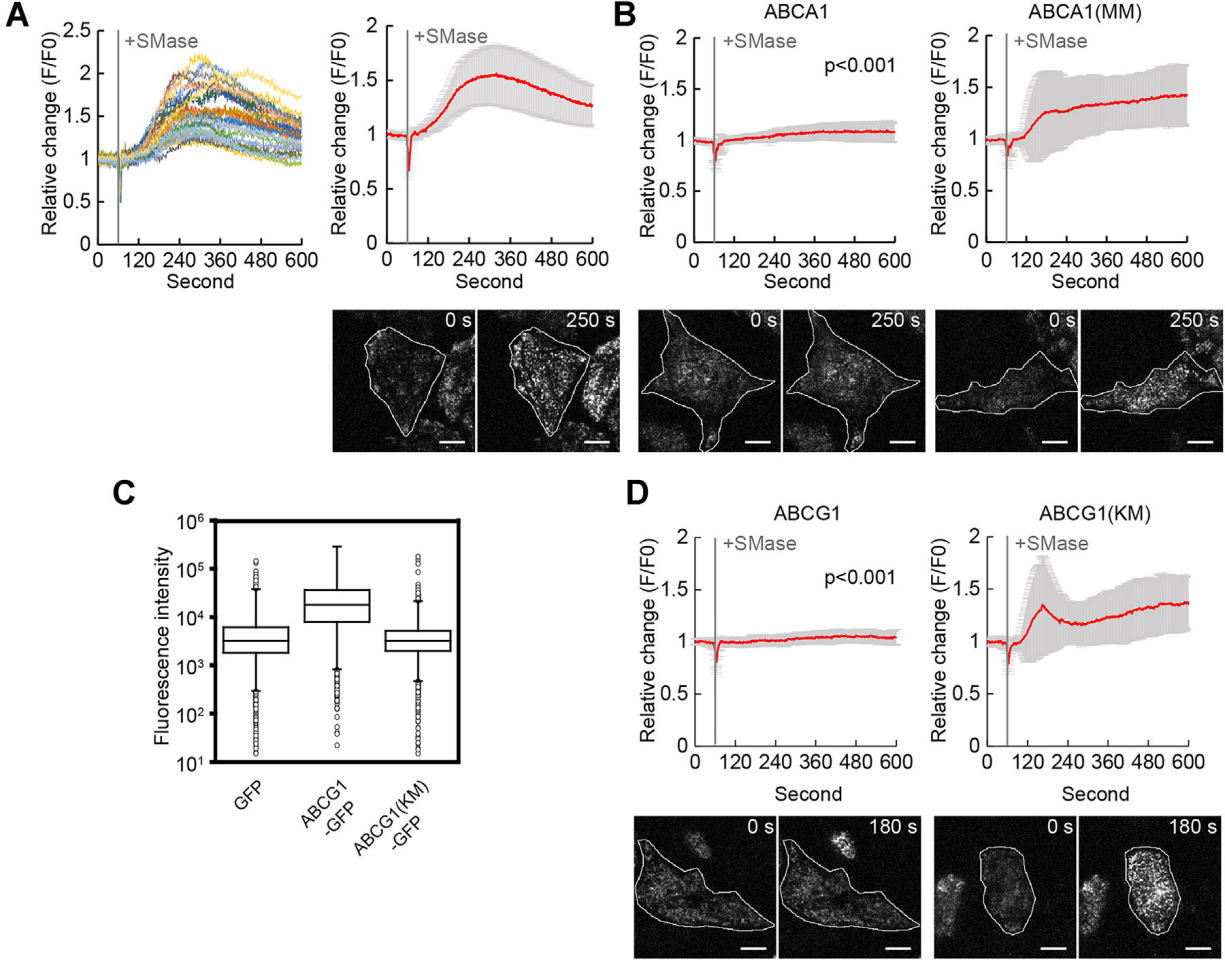
**ABCA1 and ABCG1 suppressed Aster-A recruitment after SMase treatment.***A*, HeLa/GFP-Aster-A cells were observed by TIRF microscopy. SMase was added 60 s after the imaging began. *Left*, the relative change of GFP fluorescence intensity in each cell; *right*, mean values with SD. Representative images are shown below. The *white outlines* indicate the regions of the cell. The scale bars represent 10 μm. n = 29 cells. *B*, HeLa/GFP-Aster-A cells transfected with ABCA1-mCherry or ABCA1(MM)-mCherry were observed by TIRF microscopy. SMase was added 60 s after the imaging began. Mean values are shown with SD. *p* < 0.001 *versus* ABCA1(MM)-expressing cells. Representative images are shown below. The *white outlines* indicate the regions of the cell expressing ABCA1-mCherry or ABCA1(MM)-mCherry. The scale bars represent 10 μm. The relative change in each cell is shown in Fig. S7. n = 24 to 30 cells. *C*, Alexa647-PFO-D4 binding to HeLa cells transfected with GFP, ABCG1-GFP, or ABCG1(KM)-GFP was analyzed by flow cytometry. The fluorescence intensities of Alexa647-PFO-D4 in GFP-positive cells are shown. The flow cytometry plots are shown in Fig. S8. *D*, HeLa/GFP-Aster-A cells transfected with ABCG1-mCherry or ABCG1(KM)-mCherry were observed by TIRF microscopy. SMase was added 60 s after the imaging began. Mean values are shown with SD. *p* < 0.001 *versus* ABCG1(KM)-expressing cells at 600 s. Representative images are shown below. The relative change in each cell is shown in Fig. S7. The *white outlines* indicate the regions of the cell expressing ABCG1-mCherry or ABCG1(KM)-mCherry. The scale bars represent 10 μm. n = 31 to 33 cells. PFO, perfringolysin O; SMase, sphingomyelinase; TIRF, total internal reflection fluorescence.

Sphingomyelin, which is mainly distributed at the OPM, interacts with cholesterol, but its degradation by SMase increases the IPM cholesterol level (13). Because previous reports showed that SMase treatment recruits Aster proteins to the PEcs (22, 28), we used SMase to analyze the effect of ABCA1 on Aster-A recruitment. Since the effect of SMase treatment on the Aster-A recruitment was much smaller than the effect of cholesterol loading according to a confocal microscopy analysis (Fig. S6), we used TIRF microscopy to observe changes in the Aster-A recruitment to the PEcs, as reported previously (22). GFP-Aster-A formed dot-like structures near the bottom of the cells a couple of minutes after the SMase treatment (Video S3). The mean relative fluorescence intensity reached 1.56 ± 0.28 (Fig. 6*A*). HeLa/GFP-Aster-A cells transiently expressing ABCA1 or ABCA1(MM)-mCherry were treated with SMase, and GFP-Aster-A was observed. GFP-Aster-A was not recruited to the PEcs in cells expressing ABCA1-mCherry (Video S4), and the relative fluorescence intensity at 10 min was 1.07 ± 0.10. However, in cells expressing ABCA1(MM)-mCherry, the relative fluorescence intensity was 1.43 ± 0.30 (Fig. 6*B*). These results suggest that ABCA1 suppresses Aster-A recruitment to the PEcs by flopping cholesterol to the OPM.

ABCG1, which exports cholesterol to HDL (2), also flops cholesterol and decreases the IPM cholesterol level (13, 14). To examine whether ABCG1 suppresses Aster-A recruitment to the PEcs by SMase treatment, we performed the Alexa647-PFO-D4 binding assay and TIRF microscopy. ABCG1 exports cholesterol to HDL; therefore, in the assay, FBS was removed from the medium before the transfected ABCG1 was expressed. The binding of Alexa647-PFO-D4 to cells expressing ABCG1-GFP was 6.1 times higher than in control (GFP) cells (Fig. 6*C*). In contrast, in cells expressing ABCG1(KM)-GFP, which is an ATP hydrolysis–deficient mutant, no significant change in Alexa647-PFO-D4 binding was observed. These findings suggest that ABCG1 flops cholesterol and decreases the IPM cholesterol level like ABCA1. The relative fluorescence intensity at 10 min in ABCG1-mCherry–expressing cells and ABCG1(KM)-mCherry–expressing cells was 1.04 ± 0.08 and 1.36 ± 0.25, respectively, according to the TIRF microscopy analysis (Fig. 6*D*). These results suggest that the IPM cholesterol level is the determinant of Aster-A recruitment to the PEcs, and ABCA1 and ABCG1 maintain high OPM and low IPM cholesterol levels by flopping cholesterol and suppressing Aster-A-mediated cholesterol transfer from the PM to the ER.

## Discussion

In this study, we showed that Aster-A diffuses in the ER and monitors the IPM cholesterol level by changing its length of time at the PEcs. Aster proteins have been reported to sense a transient expansion of the accessible pool of PM cholesterol *via* their GRAM domains and to facilitate the transport of this cholesterol *via* their StART-like (ASTER) domains down the concentration gradient to the ER to maintain equilibrium between accessible PM cholesterol and the ER pool (21, 22, 29). However, what exactly is accessible PM cholesterol for Aster proteins remains unclear.

The transbilayer distribution of cholesterol in the PM is still controversial. Using orthogonal lipid sensors, Liu *et al.* (13) reported that the IPM cholesterol level is 10-fold lower than the OPM cholesterol level. Steck and Lange (30) raised questions about the involvement of ABCA1 and ABCG1 because their transport activities seem insufficient to form the asymmetric distribution, which appears to use an improbable energy drain on the cell. Courtney *et al.* (31) reported that the PM cholesterol in human erythrocytes is 80% in the IPM. Erythrocytes are, however, not an ideal model for studying the cholesterol distribution in the PM because they do not have the ER, where cholesterol is synthesized and the SCAP–SREBP complex and Aster proteins function.

Very recently, Cho *et al.* (16) showed that the accessible IPM cholesterol level is much lower than the OPM cholesterol level by quantitative imaging analysis using two different types of ratiometric cholesterol sensors that are not appreciably affected by changes in the lipid environment. Some IPM cholesterol molecules are sequestered and made unavailable for lipid sensors by membrane proteins such as caveolin-1. The IPM cholesterol concentration that makes cholesterol available to cytosolic proteins, whether they are lipid sensors or signaling proteins, also applies to Aster-A. Thus, at least, the accessible IPM cholesterol level for Aster-A is much lower than the OPM cholesterol level. Moreover, in this study, we demonstrated that ABCA1 decreased the accessible IPM cholesterol level and increased the accessible OPM cholesterol level within a couple of hours (Fig. 3), suggesting that ABCA1 has adequate cholesterol flopping ability to lower, at least, the accessible IPM cholesterol level. Therefore, it is conceivable that the accessible PM cholesterol for Aster proteins refers to the accessible IPM cholesterol maintained by ABCA1.

In the ER, a cooperative response of the Scap–SREBP complex with a Hill coefficient of approximately 4 maintains ER cholesterol at 5 mol%, and the complex functions as a central switch to control PM cholesterol content in mammalian cells (18, 32). In contrast, it has been suggested that the GRAM domains of Aster proteins weakly interact with membrane cholesterol (22, 33). Because the GRAM domains can get close enough with PM cholesterol when passing through the PEcs, this weak interaction slows the diffusion rate of Aster proteins, and the IPM cholesterol concentration determines the length of time of Aster proteins staying at the PEcs. In other words, the number (*n*) of Aster molecules at the PEcs is dependent on the accessible IPM cholesterol concentration, as shown in Videos S1 and S2. Because the StART-like (ASTER) domains of Aster proteins transport cholesterol down the concentration gradient (21, 22, 29), the velocity (*v*) of cholesterol transport to the ER by an Aster molecule is also dependent on the accessible IPM cholesterol concentration when it is higher than 5 mol%. Taken together, the cholesterol transport velocity at a PEcs (*v* × *n*) will increase at an accelerating rate as accessible IPM cholesterol increases above 5 mol%. To monitor accessible IPM cholesterol constantly, it is necessary that the expression of Aster is not affected by cholesterol, which we confirmed (Fig. 1). Therefore, Aster-A may play the main role in cholesterol homeostasis in nonsteroidogenic tissues, but other Aster members may be also involved.

**Figure 7:**
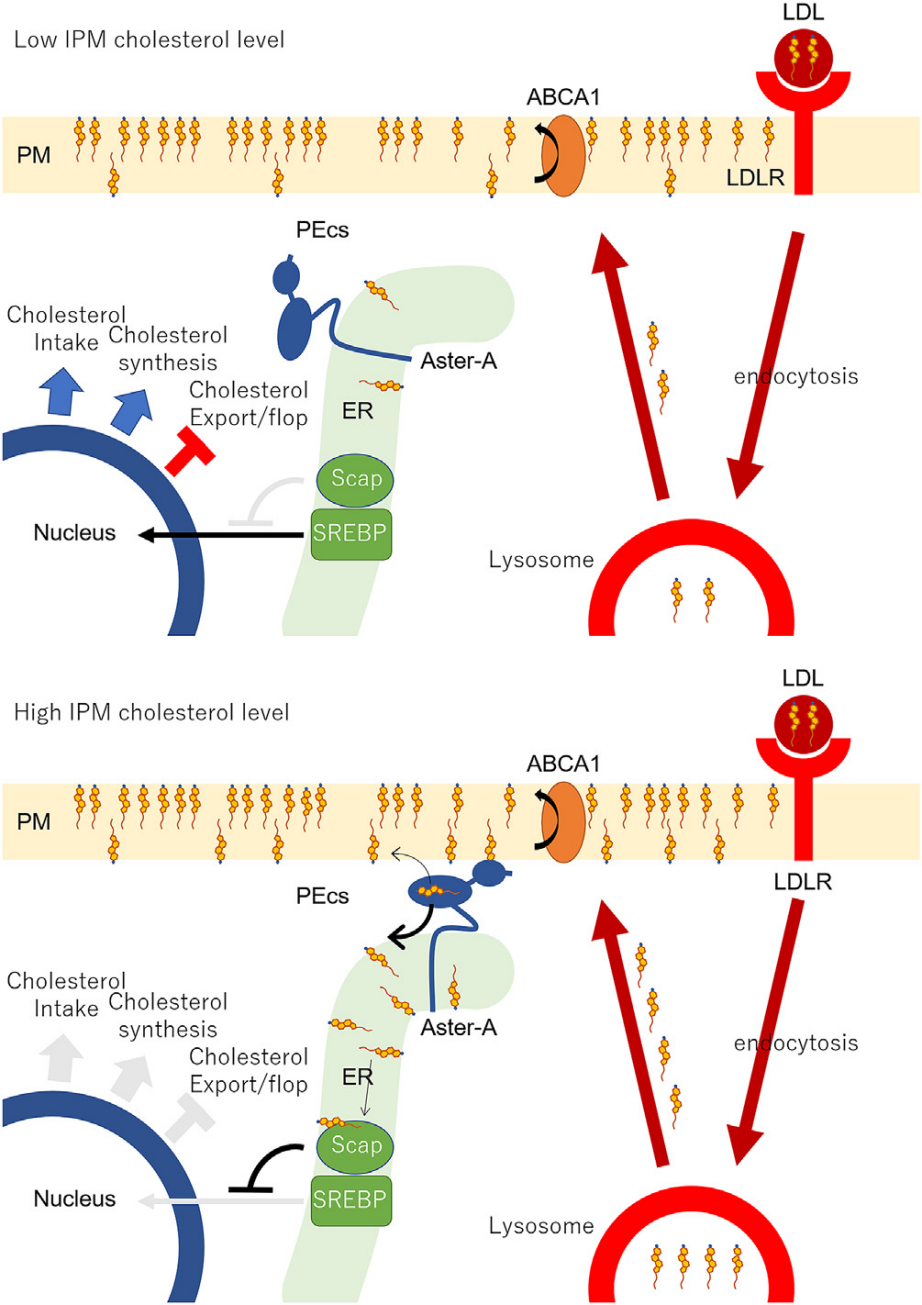
**A model depicting the mechanism for how the endoplasmic reticulum (ER) senses the plasma membrane (PM) cholesterol level.***Upper cartoon*, ABCA1 flops LDL-derived cholesterol in the PM to maintain the low cholesterol level of the inner leaflet of the PM (IPM). *Lower cartoon*, when the accessible IPM cholesterol level increases, Aster-A stays at the PM–ER contact site (PEcs) to transfer cholesterol to the ER. The SREBP–Scap system senses the increase in the ER cholesterol level, which stops the translocation of SREBP-2 to the nucleus and thus ceases cholesterol intake and synthesis. The expression of ABCA1 also increases, which flops or exports excess cholesterol, because the suppression of ABCA1 expression by microRNA *via* SREBP-2 also ceases. LDL, low-density lipoprotein; Scap, SREBP cleavage–activating protein; SREBP, sterol regulatory element–binding protein.

The SREBP–Scap system regulates cholesterol uptake, *de novo* synthesis, and export to maintain cholesterol homeostasis in cells (34, 35). When the ER cholesterol level falls below the 5 mol% threshold, SREBP-2 translocates from the ER to the nucleus to induce enzymes involved in cholesterol synthesis. SREBP-2 also induces the expression of LDL receptor. LDL that enters the cell *via* LDL receptor is degraded in lysosomes, and the bound cholesterol is delivered to the PM and then to the ER (36). The knockout of Aster proteins results in cholesterol accumulation in the PM (22), and the knockdown of Aster-A specifically slows down the response of SREBP-2 to cholesterol loading to the PM (21). Given the aforementioned, Aster-A likely regulates cholesterol homeostasis in cells by transferring LDL-derived cholesterol to the ER. In addition, it was reported that Aster proteins participate in LDL cholesterol delivery from the PM to ER (37). We, therefore, propose a mechanism for how the ER senses the PM cholesterol level as follows (Fig. 7). First, ABCA1 flops cholesterol in the PM to maintain the low accessible IPM cholesterol level. When cells receive LDL cholesterol and the accessible IPM cholesterol level locally exceeds 5 mol%, Aster-A begins to transfer cholesterol to the ER at the nearby PEcs. The SREBP–Scap system senses the increase in the ER cholesterol level, which stops the translocation of SREBP-2 to the nucleus and thus ceases cholesterol intake and synthesis. When the translocation of SREBP-2 stops, the expression of ABCA1 increases, because the suppression of ABCA1 expression by microRNA also ceases (38, 39, 40). The expression level of ABCA1 is immediately changed according to the ER cholesterol level because the turnover of ABCA1 is fast: 30 min (41). Thus, ABCA1 and Aster-A cooperatively maintain the low accessible IPM cholesterol level, and their cooperative work allows the SREBP–Scap system to monitor local and transient changes in cholesterol concentration of the whole IPM.

Taken together, our findings suggest that cellular cholesterol homeostasis depends on maintaining the accessible IPM cholesterol to be lower than ER cholesterol (5 mol%). This asymmetric cholesterol distribution also makes it possible for cholesterol to function as an intramembrane signaling molecule (13, 17, 42). Defective ABCA1 function prevents the asymmetric distribution of cholesterol. Consequently, signal transductions become dysregulated, resulting in various phenotypes including cancer progression and autoimmune activation. It is believed that mutations or a low expression of ABCA1 promote tumor development because of cholesterol accumulation (6, 43), but the disruption of the asymmetric cholesterol distribution may be also a cause. Because the amino acid sequences of ABCA1, Aster-A, and SCAP are highly conserved among mammals, birds, and fish, the mechanism to sense the PM cholesterol level and the function of cholesterol as an intramembrane signaling molecule by the asymmetric cholesterol distribution could be common among these animals. More studies are necessary to understand the physiological impact of this pathway.

## Experimental procedures

### Cell culture

HeLa cells were grown in a humidified incubator (5% CO_2_) at 37 °C in minimum essential medium (MEM) containing 10% heat-inactivated FBS. WI-38 cells and HEK293 cells were grown in a humidified incubator (5% CO_2_) at 37 °C in Dulbecco’s modified Eagle’s medium (DMEM) containing 10% heat-inactivated FBS.

### Plasmids

DNA insertion and site-directed mutagenesis were performed using an In-Fusion HD Cloning Kit (TaKaRa Bio) or by restriction enzyme fragmentation. The expression vectors for ABCA1-GFP, ABCA1(MM)-GFP, and ABCG1-GFP were generated as previously described (14). ABCG1(KM)-GFP was generated by site-directed mutagenesis with the primers 5′-GCCGGGATGTCCACGCTGATGAACATCC-3′ and 5′-CGTGGACATCCCGGCCCCGGAAGGACCC-3′. The expression vectors for mCherry-tagged ABC proteins were generated from the expression vectors for GFP-tagged ABC proteins and mCherry complementary DNA (cDNA) by an In-Fusion reaction. The expression vector for GFP-PFO-D4 was kindly provided by Dr Toshihide Kobayashi (University of Strasburg). PFO-D4 was generated as previously described (14). D4H was generated by site-directed mutagenesis using the primers 5′-TGTTTTAGATTGATAATTTCCATCCCATGTTTT-3′ and 5′-CGGACTCAGATCTCGAAGGGAAAAATAAACTTAGA-3′ and inserted into pEGFP-C2 vector (TaKaRa Bio) by the In-Fusion reaction. GFP-D4H was then inserted into pIRESpro3 vector (TaKaRa Bio) using BamHI and NheI. Aster-A cDNA (NM_020895.5) was amplified from HEK293 cDNA by PCR using the primers 5′-TGTAAGCTTTTCGACACCACACCCCACTC-3′ and 5′-AGAGAATTCTCAGGAAAAGCTGTCATCGG-3′ and was inserted into pEGFP-C3 vector (TaKaRa Bio) using EcoRI and HindIII. GFP-Aster-A was inserted into pIRESpuro3 vector using EcoRI and NheI. ΔC mutant (1–546) and Gram domain (1–256) of Aster-A were generated by the In-Fusion reaction. mCherry-KDEL was generated by the insertion of KDEL into the mCherry C terminus and a signal peptide into the N terminus by the In-Fusion reaction.

### Transfection and stable cell lines

For transient expressions, cells were transfected with 1 μg/ml of each expression vector using 2 μg/ml Polyethyleneimine “MAX” (PolySciences) (44) in a culture medium containing 10% FBS. For stable expressions, cells were transfected with 1.25 μg/ml of each expression vector using Lipofectamine LTX (Thermo Fisher Scientific) in a culture medium containing 10% FBS and selected with 0.5 μg/ml puromycin for a couple of weeks. HeLa/GFP-D4H were cloned after the selection.

### Protein analysis

Cells were lysed with 0.1% Triton in PBS without CaCl_2_ or MgCl_2_ supplemented with EDTA-free protein inhibitor cocktail (cOmplete; Roche) on ice. For ABCA1, proteins were diluted in sampling buffer (5 mM Tris–HCl, 40 mM DTT, 2% SDS, 1 mM EDTA, 1% sucrose, and 0.01 mg/ml pyroninY), heated at 50 °C for 10 min, diluted again in sampling buffer supplemented with 5 M urea, and electrophoresed on 5 to 20% PAGEL (ATTO). All other proteins were diluted in Laemmli buffer (45), heated at 98 °C for 5 min, and electrophoresed on 5 to 20% PAGEL (ATTO). The proteins were transferred to an Immobilon-P Transfer Membrane (Merck), blocked with 10% Blocking One (Nacalai Tesque, Inc), and blotted with the indicated primary antibody. The anti–extracellular domain of ABCA1 (MT-25) was generated as previously described (15). Anti-Aster-A antibody (catalog no.: NBP2-32148; Novus Biologicals), antivinculin antibody (catalog no.: V9131-2Ml; SIGMA), and anti-GFP antibody (catalog no.: sc-9996; Santa Cruz) were purchased. Goat antimouse immunoglobulin G (IgG) (H + L) or goat anti-rabbit IgG (H + L) (Bio-Rad) were used as secondary antibodies. The immune signal was visualized using immunoStar Zeta or LD (WAKO).

### Quantitative PCR

The total RNA of cells was purified with an RNeasy kit (QIAGEN) and reverse transcribed to cDNA with PrimeScript RT Master Mix (TaKaRa). Quantitative PCR was performed with the primers listed in Table S2 and THUNDERBIRD SYBR qPCR Mix (TOYOBO). *hrps18* was used as the internal control.

### Imaging

For the confocal microscopy imaging of GFP-Aster-A, HeLa/GFP-Aster-A cells were plated in a glass-base dish (IWAKI) coated with fibronectin in MEM containing 10% FBS and incubated overnight. When transfected with ABCA1 or ABCA1(MM) (Fig. 5*A*), the cells were plated in a 6-well plate, transfected with the indicated vectors in MEM containing 10% FBS and 2.5 μM PSC-833, and reseeded to a glass-base dish coated with fibronectin on the following day. After the cells were attached sufficiently, they were incubated in MEM supplemented with 0.02% bovine serum albumin (BSA) for 2 h to activate ABCA1 cholesterol–flopping activity. The cells were then treated with MβCD–cholesterol complex at the indicated concentrations or 0.2 U/ml SMase (catalog no.: s7651-50UN; SIGMA) for 5 min and fixed with 4% paraformaldehyde. To prepare 4 mM MβCD–cholesterol complex, 4 mM cholesterol and 36 mM MβCD were mixed in PBS and incubated at 60 °C overnight. Because ABCA1 localizes not only on the PM but also on endosomes, the expression level of ABCA1 on the PM was measured using the anti–extracellular domain of ABCA1 antibody (MT-25) with no permeabilization. The cells were blocked with 10% goat serum (SIGMA) for 30 min and stained with MT-25 and Alexa555-conjugated goat antimouse IgG (H + L) (Thermo Fisher Scientific) for 1 h. The PM was stained with CellMask Deep Red for 10 s before observation. Imaging was performed with an LSM 700 confocal microscope equipped with α Plan-Apochromat 63×/1.40 Oil DIC M27 objective lens (Carl Zeiss).

To image the internalization of TopFluor-cholesterol, HeLa/Aster-A cells were plated in a glass-base dish coated with fibronectin, transfected with ABCA1-mCherry in MEM containing 10% FBS and 5 μM PSC-833, and incubated overnight. The cells were then incubated in FluoroBrite DMEM (Thermo Fisher Scientific) supplemented with 0.02% BSA, sodium pyruvate, and GlutaMAX (Thermo Fisher Scientific) for 1.5 h and with anti–Na^+^/K^+^ ATPase β3 subunit antibody (ECM Biosciences) and Alexa633-conjugated goat antimouse IgG (H + L) for 30 min, and treated with 0.2 mM MβCD–cholesterol complex mixed with TopFluor-cholesterol for 5 min. Imaging was performed at 37 °C under 5% CO_2_ with the LSM 700 confocal microscope equipped with the aforementioned lens.

For high-resolution imaging, HeLa/GFP-Aster-A cells were plated in a glass-base dish coated with fibronectin, transfected with mCherry-KDEL, and incubated in MEM containing 10% FBS overnight. The cells were treated with MβCD–cholesterol complex at the indicated concentrations in FluoroBrite DMEM supplemented with 0.02% BSA, sodium pyruvate, and GlutaMAX. Imaging was performed at 37 °C under 5% CO_2_ with an LSM 880 confocal microscope equipped with α Plan-Apochromat 100×/1.46 Oil DIC M27 Elyra objective lens and an Airyscan detector (Carl Zeiss). Images were Airyscan processed automatically using Zeiss Zen2 software.

For TIRF imaging, HeLa/GFP-Aster-A cells were plated on a glass-base dish coated with fibronectin, transfected with the indicated vectors, and incubated for 6 h. The medium was then exchanged to MEM supplemented with 0.02% BSA and incubated overnight. Imaging was performed at 37 °C under 5% CO_2_ with an ECLIPSE Ti TIRF microscope equipped with an Apo TIRF 60xC Oil objective lens. Sixty seconds after beginning the video recording, the cells were treated with 0.2 U/ml SMase in MEM without phenol red (Thermo Fisher Scientific) supplemented with 0.02% BSA and GlutaMAX. The frame rate was set to one image per second.

### Image processing and calculation

Images were processed using Fiji software  (National Institutes of Health) to calculate the ratio of the GFP-Aster-A fluorescence intensity on the PM to that of the total cell area. A region of the PM was detected from images positive for CellMask Deep Red, and a region of the total cell was detected from the images positive for GFP-Aster-A or CellMask Deep Red. The GFP intensities on the PM and in the total cell were measured. When ABCA1 was expressed, the mean ABCA1 intensity on the PM was measured, and the autofluorescence of the HeLa parent cells was subtracted as background.

To calculate the relative change in the GFP-D4H fluorescence intensity, a region of the cell positive for ABCA1-mCherry was detected from each image. Background was removed by the subtract background command in Fiji software, and the GFP intensity was measured.

To calculate the relative change in the GFP-Aster-A fluorescence intensity, a region of the cell was manually selected. Background was removed by the subtract background command in the Fiji software, and the GFP intensity was measured.

### Flow cytometry analysis

PFO-D4 was purified and labeled with Alexa Fluor 647 as previously described (14). For the PFO-D4 binding assay, HeLa cells were plated in a 12-well plate, transfected with the indicated vectors in MEM containing 10% FBS and 5 μM PSC-833, and incubated overnight. The cells were then incubated in MEM supplemented with 0.02% BSA for 2 h. The collected cells were incubated with 0.125 μg/ml Alexa647-PFO-D4 in Hanks' balanced salt solution (HBSS) at 20 °C for 30 min and analyzed with an Accuri C6 flow cytometer (BD). The data were exported to Excel and divided into GFP negative and positive cells at the indicated fluorescence intensities. The percentages of the plots and the median fluorescence intensities of Alexa647-PFO-D4 were calculated. Pseudocolor plots were generated using Cytospec software (Purdue University Cytometry Laboratories) for each sample, and 30,000 cells were analyzed.

For the TopFluor-cholesterol assay, HeLa/Aster-A and HEK293 cells were transfected with ABCA1 in medium containing 10% FBS and 5 μM PSC-833. The cells were incubated in serum-free medium for 2 h and treated with 0.2 mM MβCD–cholesterol complex mixed with TopFluor-cholesterol for 5 min. The collected cells were incubated with the anti–extracellular domain of ABCA1 antibody (MT-25) and Alexa555-conjugated second antibody in HBSS at 20 °C for 30 min and analyzed with the Accuri C6 flow cytometer. Pseudocolor plots were generated using Cytospec software.

### Measurement of sphingomyelin

HeLa/GFP-Aster-A cells were plated in a 24-well plate. On the following day, the cells were treated with SMase at the indicated concentrations for the indicated times and trypsinized. Lipids in the cells were extracted with chloroform and methanol (2:1) and dissolved in HBSS supplemented with 0.1% Triton X-100 and 5 mM cholic acid. About 50 μl of the lipid solution was then added to a 96-well black plate and mixed with an equal volume of enzyme mixture solution (0.4 U/ml SMase, 60 U/ml calf intestinal alkaline phosphatase, 0.4 U/ml choline oxidase, 40 mU/ml horseradish peoxidase, and 50 μM AmplexUltraRed (Thermo Fisher Scientific) in HBSS. After incubation at 37 °C for 30 min, the fluorescence intensity of AmplexUltraRed was measured with a microplate reader (Cytation 5; BioTek).

### Statistical analysis

The statistical significance of differences between mean values was evaluated using the unpaired *t* test. Multiple comparisons were evaluated using the Tukey test following one-way ANOVA. The exact *p* values are listed in Table S1. All experiments were performed at least twice.

## Data availability

All data are contained within the article.

## Supporting information

This article contains supporting information.

## Conflict of interest

The authors declare that they have no conflicts of interest with the contents of this article.

## Supporting information

Supplemental Figures S1–S8 and Video Captions
