# Supplementary material for: ABCA1 and cholesterol transfer protein Aster-A promote an asymmetric cholesterol distribution in the plasma membrane
Source: J Biol Chem. 2022 Nov 14;298(12):102702. doi: 10.1016/j.jbc.2022.102702 (PMC9747601; doi:10.1016/j.jbc.2022.102702)
Supplement: Supplemental Figures S1–S8 and Video Captions [file mmc1.docx]

ATP-binding cassette A1 and cholesterol transfer protein Aster-A promote an asymmetric cholesterol distribution in the plasma membrane

Fumihiko Ogasawara and Kazumitsu Ueda*

Supplemental Figures S1–S8, Tables S1, S2, and Videos S1-S4


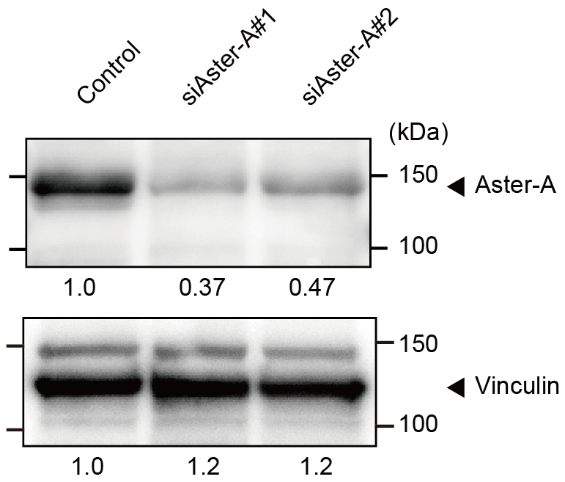


Figure S1. The specificity of the anti-Aster-A antibody was confirmed by knockdown experiments using siRNAs.

WI-38 cells were transfected with siRNAs and incubated for 24 h. The expressions of Aster-A and vinculin, a loading control, were analyzed by western blotting. Band intensities relative to the control are shown below each band.


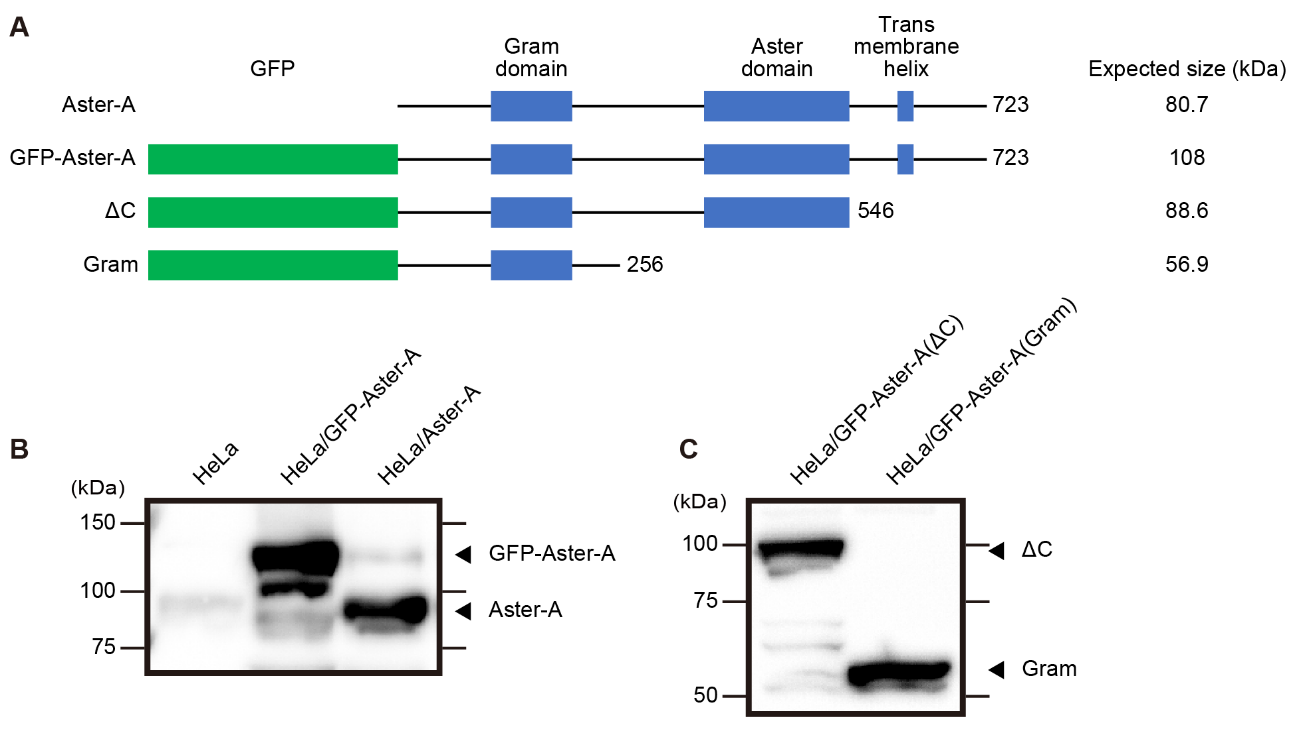


Figure S2. GFP-Aster-A was correctly expressed.

A. A schematic representation of Aster-A, GFP-Aster-A, GFP-Aster-A(ΔC), and GFP-Aster-A (Gram). B. The expression of Aster-A and GFP-Aster-A in HeLa cells, HeLa/GFP-Aster-A, and HeLa/Aster-A cells were analyzed by western blotting. C. ΔC mutant (1-546) and Gram domain (1-256) of Aster-A were transfected into HeLa cells, and their expressions were analyzed by western blotting with anti-GFP antibody.


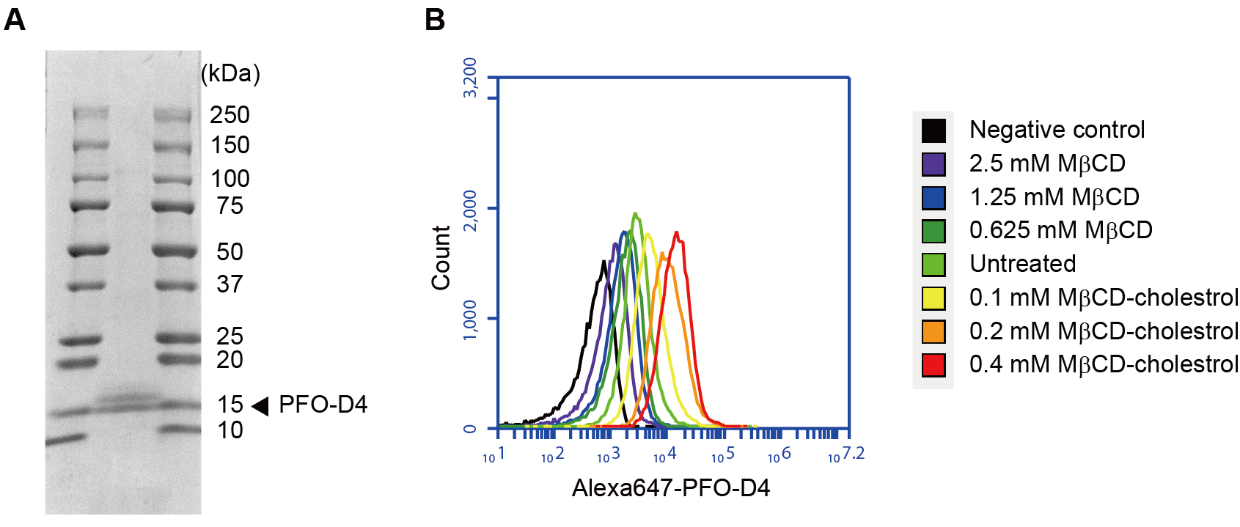


Figure S3. Alexa647-PFO-D4 binds to the cell surface with increasing cholesterol levels at the plasma membrane.

A. The size of Alexa647-PFO-D4 was confirmed by Coomassie staining. The lower and upper bands correspond with PFO-D4 and PFO-D4 labeled with a Alexa647 molecule, respectively. B. HeLa cells were treated with 0, 0.625, 1.25, and 2.5 mM MβCD or 0.05, 0.1, 0.2, and 0.4 mM MβCD-cholesterol and analyzed by FACS with Alexa647-PFO-D4. Cells not treated with Alexa647-PFO-D4 are shown as a negative control.


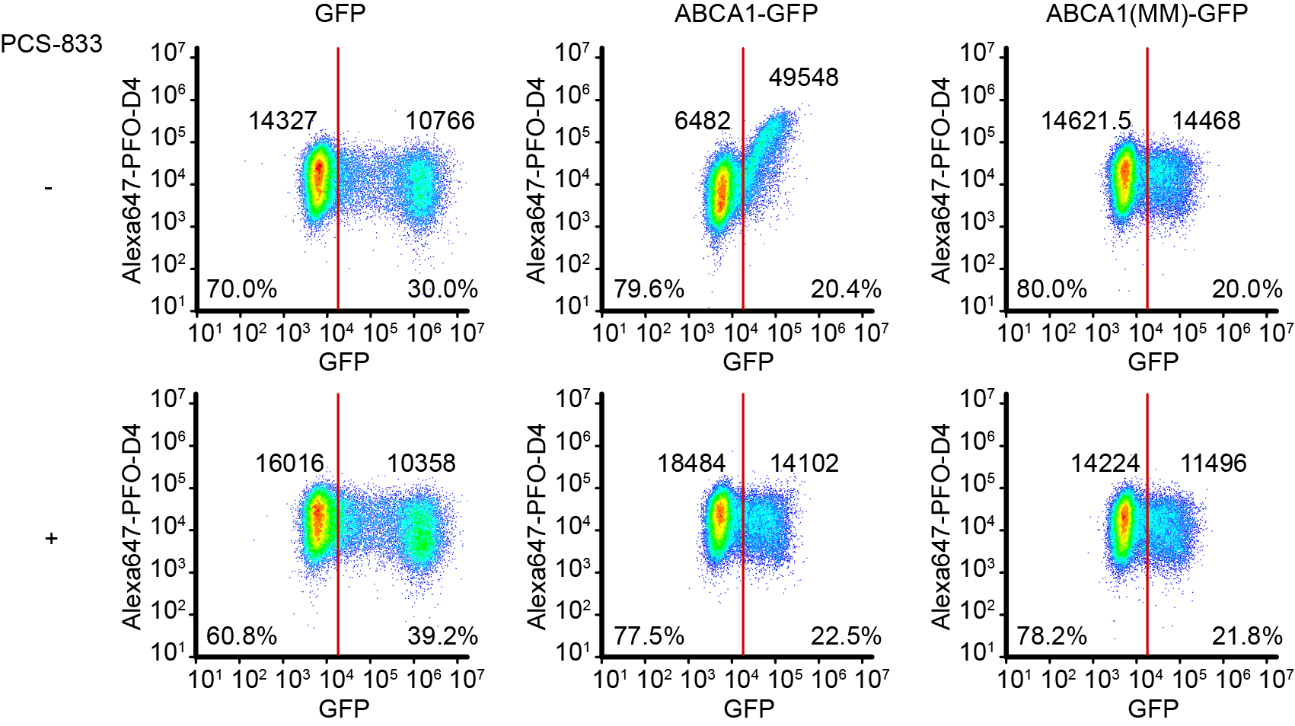


Figure S4. Flow cytometry plots.

Flow cytometry plots of the data shown in Fig. 3A. The plots were divided into GFP negative and positive cells at a fluorescence intensity of 17,783, and the percentage of the plots and median fluorescence intensities of Alexa647-PFO-D4 are shown.


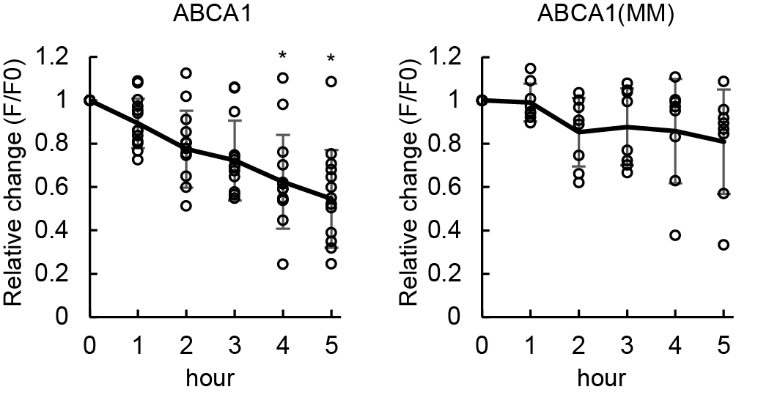


Figure S5. The same assay as Fig. 3B,C with another clone of HeLa/GFP-D4H cells.

The solid line indicates mean values. Error bars indicate S.D. *p<0.05 vs. ABCA1(MM)-expressing cells. n=8-14 cells.


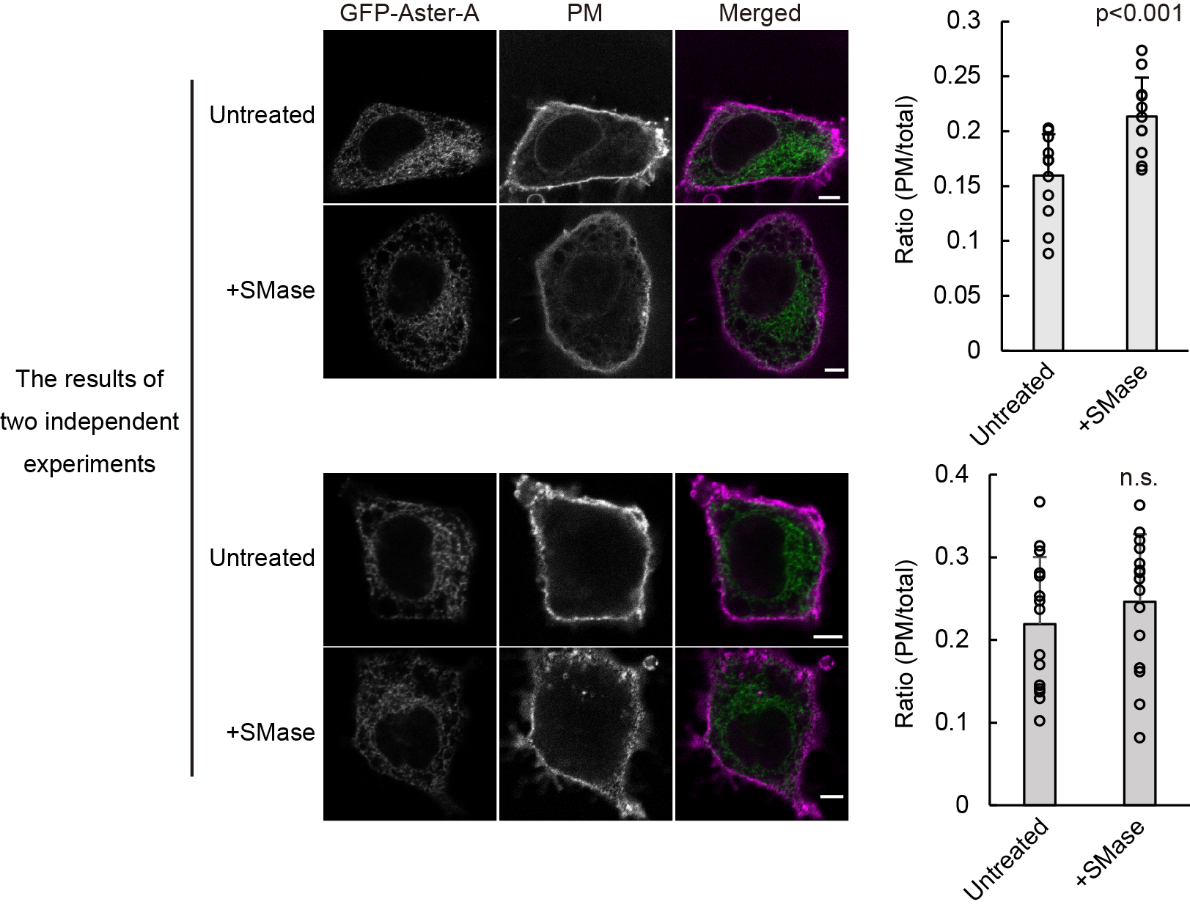


Figure S6. The effect of SMase on Aster-A recruitment to the PEcs was not always significant.

HeLa/GFP-Aster-A cells were treated with or without SMase in serum-free medium for 5 min, fixed with 4% paraformaldehyde, and observed by confocal microscopy. The PM was stained with CellMask Deep Red. The upper and lower data show the results of two independent experiments. The data of untreated cells in Figure 2A is reused in the upper data. The ratio of GFP Aster-A on the PM to that in the total cell area is shown. Mean values are shown with S.D. The ratio tended to increase by SMase treatment. Scale bars, 5 µm. p < 0.001 vs. untreated. n.s., not significant. n=11-15 cells.


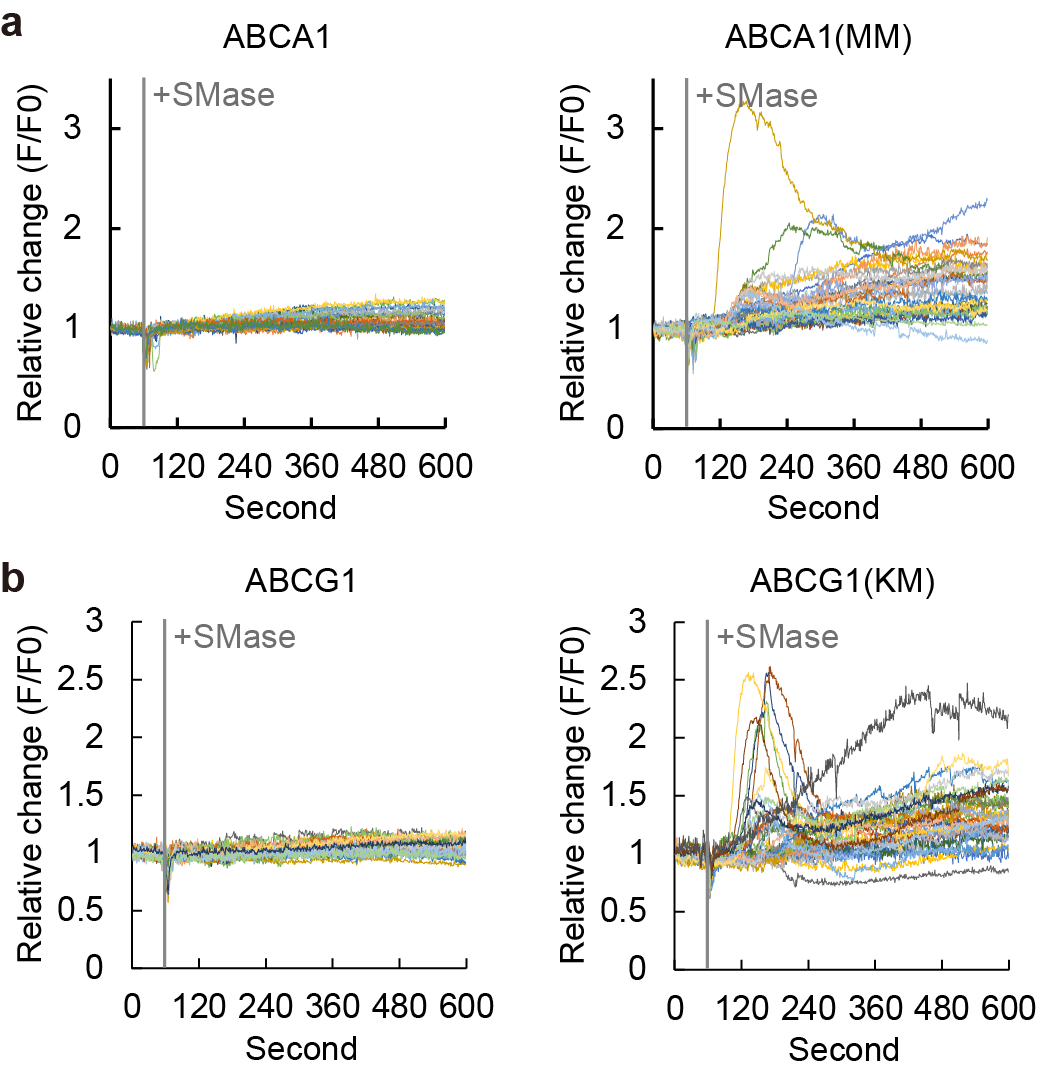


Figure S7. Relative change of the fluorescence intensity for each cell in Figure 6.


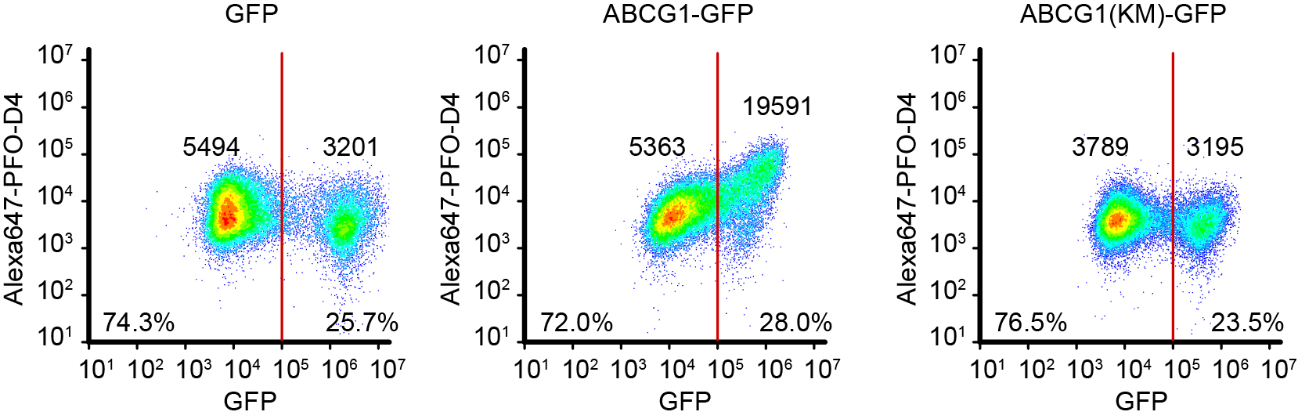


Figure S8. Flow cytometry plots.

Flow cytometry plots of the data shown in Figure 6b. The plots were divided into GFP negative and positive cells at a fluorescence intensity of 100,000, and the percentage of the plots and median fluorescence intensities of Alexa647-PFO-D4 are shown.

Video S1. GFP-Aster-A movement after cholesterol loading.

HeLa/GFP-Aster-A cells were treated as described in the legend of Fig. 2. The movie begins about 30 s after the cholesterol loading. Green, GFP-Aster-A. Magenta, ER marker (mCherry-KDEL).

Video S2. GFP-Aster-A movement after cholesterol loading at a low concentration (0.1 mM).

The movie begins about 30 s after the cholesterol loading. Green, GFP-Aster-A. Magenta, ER marker (mCherry-KDEL).

Video S3. GFP-Aster-A movement after SMase treatment visualized by TIRF microscopy.

HeLa/GFP-Aster-A cells were treated with SMase at 60 s.

Video S4. GFP-Aster-A movement in cells transfected with ABCA1-mCherry after SMase treatment visualized by TIRF microscopy.

HeLa/GFP-Aster-A cells were treated with SMase at 60 s. White arrowheads show cells expressing ABCA1-mCherry.
