# Supplementary material for: ABCA1 and cholesterol transfer protein Aster-A promote an asymmetric cholesterol distribution in the plasma membrane
Source: J Biol Chem. 2022 Nov 14;298(12):102702. doi: 10.1016/j.jbc.2022.102702 (PMC9747601; doi:10.1016/j.jbc.2022.102702)
Supplement: Supplemental Table S1 [file mmc2.pdf]

| | | group1 | group2 | p value |
|---|---|---|---|---|
| Figure 1B | ABCA1 | 0.02% BS/ | 10% FBS | 1.38E-06 |
| | | 0.02% BS/ | T0 | 0 |
| | | 0.02% BS/ | 25OHC | 3.07E-05 |
| | Aster-A | 0.02% BS/ | 10% FBS | 0.448 |
| | | 0.02% BS/ | T0 | 6.59E-02 |
| | | 0.02% BS/ | 25OHC | 0.302 |
| Figure 2B | | Untreated | +Choleste | 4.05E-13 |
| Figure 3C | 1 h | ABCA1 | ABCA1(M | 3.54E-05 |
| | 2 h | ABCA1 | ABCA1(M | 3.67E-08 |
| | 3 h | ABCA1 | ABCA1(M | 2.57E-11 |
| | 4 h | ABCA1 | ABCA1(M | 5.15E-13 |
| Figure 4C | | 0 U/mL | 0.2 U/mL | 9.11E-05 |
| | | 0 U/mL | 1.0 U/mL | 2.77E-05 |
| | | 0 U/mL | 0.2 U/mL | 1.25E-05 |
| | | 0 U/mL | 1.0 U/mL | 1.66E-05 |
| Figure 6B | | ABCA1 | ABCA1(M | 4.18E-07 |
| Figure 6D | | ABCG1 | ABCG1(K | 4.67E-08 |
| Figure S6 | 1 h | ABCA1 | ABCA1(M | 5.09E-02 |
| | 2 h | ABCA1 | ABCA1(M | 3.08E-01 |
| | 3 h | ABCA1 | ABCA1(M | 6.84E-02 |
| | 4 h | ABCA1 | ABCA1(M | 2.91E-02 |
| | 5 h | ABCA1 | ABCA1(M | 1.82E-02 |
| Figure S7 | | Untreated | +SMase | 2.04E-03 |
| | | Untreated | +SMase | 3.75E-01 |
