# Supplementary material for: ABCA1 and cholesterol transfer protein Aster-A promote an asymmetric cholesterol distribution in the plasma membrane
Source: J Biol Chem. 2022 Nov 14;298(12):102702. doi: 10.1016/j.jbc.2022.102702 (PMC9747601; doi:10.1016/j.jbc.2022.102702)
Supplement: Supplemental Table S2 [file mmc3.pdf]

hrps18	gatgggcggcggaaa
	cggcccacacccttaatg
abca1	aacagtttgtggcccttttg
	agttccaggctggggtactt
gramd1a	agaccacgatctccatccag
	tttcaagcagtgcattctgc
